# Exploring Neural Markers of Reward and Loss Processing and Problematic Parenting Styles A Mothers With and Without Histories of Depression

**DOI:** 10.1002/dev.70083

**Published:** 2025-09-10

**Authors:** Jennifer H. Suor, Rebecca Mueller, Stewart A. Shankman, Katie L. Burkhouse

**Affiliations:** ^1^ Department of Psychiatry University of Illinois Chicago Chicago Illinois USA; ^2^ Department of Psychiatry and Behavioral Sciences Northwestern University Feinberg School of Medicine Chicago Illinois USA; ^3^ Department of Psychology The Pennsylvania State University University Park Pennsylvania USA

**Keywords:** event‐related potentials, feedback negativity, maternal depression, parenting styles, reward positivity

## Abstract

Depressed mothers often experience parenting difficulties, which can persist after their symptoms have remitted. However, not all depressed mothers show parenting struggles, suggesting that there could be unidentified characteristics that increase risk. Specifically, neurobiological models emphasize that reward system deficits contribute to maladaptive parenting and depression, but no studies have evaluated how they could conjointly lead to parenting challenges. This study focused on event‐related potential (ERP) components, the reward positivity (RewP), and feedback negativity (FN), which assess neural responsiveness to reward and loss feedback, respectively. Mothers with (*n* = 81) and without (*n* = 55) depression histories completed a monetary reward task to elicit the FN and RewP, and depression and parenting questionnaires. We found mothers demonstrating a blunted FN to loss and increased depressive symptoms reported greater authoritarian parenting, whereas there was no association between depressive symptoms and authoritarian parenting among mothers exhibiting greater neural loss responsiveness. Furthermore, these effects were specific to maternal current depressive symptoms and not major depressive disorder (MDD) diagnostic history. Maternal depressive symptoms were associated with reduced warmth, but the RewP did not moderate this association. Together, findings suggest that depressed mothers with blunted responsiveness to negative cues may be particularly vulnerable to adopt authoritarian parenting styles.

## 1 Introduction

Maternal depression is highly prevalent, with estimates that 1 in 10 mothers experience depression annually (e.g., Ertel et al. 2011), which is a significant public health concern. Maternal depression confers negative developmental consequences for offspring across childhood and adolescence (Goodman and Gotlib 2002, 1999). For example, children of depressed mothers are two to five times more likely to develop a psychological disorder and three times more likely to develop depression than children whose mothers are not depressed (Weissman et al. 2016, 2006). Maternal depression is also the most potent predictor of paternal depression, underscoring its potential negative impact on all family members (Gutierrez‐Galve et al. 2019, 2015).

Crucially, in the majority of societies, mothers are the primary caregivers of their children and are more likely to be single parents than fathers, particularly among racioethnic minorities (Cairney et al. 2003; Callaghan et al. 2024). This is important given a key mechanism through which maternal depression predicts socioemotional challenges in offspring is negative parenting behaviors (e.g., Foster et al. 2008; Frye and Garber 2005; Goodman et al. 2011). However, not all mothers with histories of depression exhibit maladaptive parenting suggesting the importance of identifying potential moderating factors that increase risk for parenting difficulties in some mothers with depression. There is an emerging body of research that has suggested that mothers who exhibit atypical neuroaffective processing styles demonstrate less sensitivity during parent–child interactions (Hajal and Loo 2021). However, research in this area is still very limited. Furthermore, many of the studies that do exist have focused on mothers with infants and have not examined mothers with preadolescent and adolescent offspring (Hajal and Loo 2021). This gap is significant as parenting roles change substantially during different stages of child development. For instance, parents of adolescents face the unique task of being emotionally responsive to their teenager's increasingly complex socioemotional needs and determining how to implement appropriate behavioral control strategies that still support their adolescent's ability to make their own decisions and form their own identities (Steinberg 2000; Steinberg et al. 2002). Moreover, there are normative increases in conflict in the parent–child relationship during the adolescent years (Steinberg 2000; Steinberg et al. 2002). Accordingly, identifying the parental neural mechanisms that contribute to parenting challenges during older stages of child development when nonoptimal parenting is known to increase risk for mental health and other psychosocial impairments could improve the effectiveness of family‐based preventive interventions.

### 1.1 Maternal Depression and Parenting Styles

Intergenerational depression risk models propose that negative parenting is a key mechanism through which risk for depression is increased among offspring (Forman et al. 2007; Goodman and Gotlib 2002). Negative parenting behaviors and styles are defined as those that are unresponsive to children's emotional and physical needs, low in warmth and nurturance, and overly controlling of their behavior (Baumrind 1971; Darling and Steinberg 2017). For example, many studies have found that depressed mothers exhibit lower levels of sensitivity, responsiveness, and warmth toward their children than nondepressed mothers (Foster et al. 2008; Lovejoy et al. 2000). Prior research has also found that depressed mothers display more aggravation and negative emotions toward their children and report lower feelings of investment in them (Chad‐Friedman et al. 2024; Conners‐Burrow et al. 2014; Dougherty et al. 2013). Crucially, the negative impacts of depression on parenting are also observed among mothers with subclinical levels of depression who are often missed by screenings in primary care settings (e.g., Conners‐Burrow et al. 2014; Trussell et al. 2018).

Prior work has also demonstrated that mothers with current and lifetime histories of depression often display problematic parenting styles. The term parenting style refers to parent's set of beliefs, values, and practices that shape the milieu of the parent–child relationship (Darling and Steinberg 2017). Specifically, studies have found that some mothers with depression exhibit authoritarian parenting styles (Goodman 2020), which is characterized by an approach to parenting that is low in responsiveness to the socioemotional needs of children and high in behavioral control (e.g., overcontrolling behavior, limited independence, and the belief of complete compliance to parental rules and expectations) (Baumrind 1971; Darling and Steinberg 2017). Authoritarian parenting styles have been linked to poor parent–adolescent relationship quality, unhealthy communication patterns, and negative adolescent socioemotional outcomes, such as depression, low self‐esteem, conduct problems, and risk taking (Chad‐Friedman et al. 2024; Hart et al. 2019; Heaven and Ciarrochi 2008; King et al. 2016; Smetana 2017).

Despite established links between maternal depression and parenting challenges, meta‐analytic studies are increasingly finding that effect sizes between maternal depression and parenting are small to medium (*d*s = 0.16–0.40), differ based on parenting behaviors and styles examined (Lovejoy et al. 2000), and demonstrate significant heterogeneity and inconsistency across studies (e.g., Goodman et al. 2020. In light of this, there has been a recent call from researchers to consider different moderating characteristics that could increase or buffer against the negative impact of maternal depressive symptoms on parenting (e.g., Goodman 2020). Relatedly, researchers have also argued for evaluating these associations in community samples of mothers whose depressive symptoms are likely more representative of mothers who experience depression compared to small clinical samples where mothers exhibit atypical levels of depression severity (Hammen and Brennan 2003). Taking a dimensional approach to maternal depression and parenting patterns has the potential to increase nuance in conceptual models and improve identification of a larger group of mothers and their children in need of support who would otherwise go undetected if they did not meet full diagnostic criteria for major depressive disorder (MDD).

### 1.2 Event‐Related Potential (ERP) Components and Parenting Styles

Parenting practices and styles are influenced by multiple neurobiological and physiological systems supporting attention, motivation, affect, and cognition; and functioning in these systems plays an important role in organizing parenting behaviors in response to different types of parent–child interactions (Barrett and Fleming 2011; Hajal and Loo 2021; Mileva‐Seitz and Fleming 2011). For example, parents are biologically wired to experience interactions with their offspring as rewarding and are motivated to build strong, positive bonds with their children as survival of their offspring depends on it (Barrett and Fleming 2011). This requires an ability for parents to flexibly adapt their attention, emotions, and behavior to meet the needs of their children at different developmental stages and in a manner that is appropriate to broader contextual characteristics (e.g., social support, security, and environmental resources).

Most of the research on biological processes implicated in parenting has been conducted with mothers of infants and young children. This body of work has largely focused on how maternal neurobiology changes across pregnancy and postpartum to facilitate the transition to parenthood (Barrett and Fleming 2011; Callaghan et al. 2024), as well as how cognitive and affective neural mechanisms, financial stressors, and mental health challenges could additively contribute to problematic parenting among parents who have young children (Deater‐Deckard et al. 2012; Moses‐Kolko et al. 2014; Pawluski et al. 2017). For example, some recent studies have found that mothers with executive function challenges exhibit harsher disciplinary practices and lower sensitivity with their preschool‐age children, with these effects particularly pronounced among mothers with other social cognitive processing risk characteristics (e.g., dysfunctional attributions about child misbehavior) or living in chaotic and under‐resourced environments (Deater‐Deckard, Chen, et al. 2012; Deater‐Deckard, Wang, et al. 2012; Deater‐Deckard and Bell 2017; Sturge‐Apple et al. 2017, 2014). Related studies have documented greater autonomic reactivity and harsher parenting in other vulnerable groups of mothers, such as those with histories of maltreatment and with lower incomes (Li et al. 2022; Reijman et al. 2016). This work highlights how atypical neurocognitive patterns might additively contribute to parenting challenges across different at‐risk groups of parents.

Using electrophysiology and neuroimaging, an accumulating and promising body of research has found evidence of atypical patterns in affective and reward processing among mothers who report less sensitivity during parent–child interactions and maladaptive parenting styles (Hajal and Loo 2021; Levinson et al. 2017, 2022). For instance, using electroencephalography (EEG), a few studies have examined ERPs indexing affective processing to understand relations with parenting styles. Two recent studies have focused on the reward positivity (RewP) component, which is an upward positive inflection in the waveform that is elicited by receiving reward feedback. Neuroimaging studies suggest that the RewP is generated by the striatum, the primary reward center in the brain (e.g., Proudfit 2015). Levinson et al. (2017) found that parents of adolescent children who rated themselves as more authoritarian and permissive in their parenting style demonstrated a reduced observational RewP, which they measured by examining ERPs while parents watched their children win and lose money. They concluded that parents who use more controlling or indulging parenting practices respond less positively to offspring outcomes. In a subsequent study, Levinson et al. (2022) examined these questions in a sample of mothers with young children and included some mothers who had histories of depression. In this follow‐up study, they found that a reduced observational RewP was more predictive of a permissive parenting style compared to an authoritarian parenting style.

Monetary gain tasks, such as those employed by Levinson and colleagues, also elicit the feedback negativity (FN), an ERP that is a negative deflection in the waveform that shows increased negativity in response to loss or non‐reward feedback (Hajcak et al. 2006; Holroyd et al. 2006). Although not the focus of their study, Levinson et al. (2002) observed a stronger bivariate correlation between authoritarian parenting style and observational FN compared to observational RewP. Differential effects are not entirely unexpected given that FN and RewP are produced by distinct neural generators in the anterior cingulate cortex (ACC) and ventral striatum, respectively (Gehring and Willoughby 2002; Miltner et al. 1997; Proudfit 2015). For example, utilizing time frequency analyses, studies have demonstrated that the RewP is associated with gain‐related increases in delta‐band activity in the basal ganglia, and FN is related to loss‐related increases in theta‐band activity in the ACC (Bernat et al. 2008, 2011, 2015; Foti et al. 2015). Neuroimaging studies also find that distinct neural circuits underlie reward and punishment cues, with the lateral orbital frontal cortex (OFC), insula, and ACC acting as the key regions that support punishment‐related processes and subsequent behavioral adjustments (Gottfried and Dolan 2004; O'Doherty et al. 2003; Wrase et al. 2007). Similarly, ERP studies have found that the FN in response to loss cues might reflect an action monitoring system that detects conflict between action and expected rewards and provides a neural signal to adjust behavior to achieve desired outcomes after negative feedback (Band et al. 2009; Bernat et al. 2015; Webb et al. 2016). However, there have not been studies that have explicitly delineated the potential differential roles of RewP and FN in parenting styles among depressed mothers with older children and if altered RewP and FN patterns might conjointly contribute to parenting challenges among some mothers with depression compared to others. Research that evaluates the role of neuroaffective risk mechanisms in maternal depression and parenting models is important for increasing understanding of the heterogeneity in parenting among mothers with histories of depression and enhancing our ability to identify and optimally support subgroups of mothers that are at particularly high risk for maladaptive parenting.

Accordingly, the purpose of this present study was to expand upon prior work in several important ways. The current study sought to evaluate potential interactions between neural markers of reward and loss sensitivity and maternal depression in their relation to authoritarian and warm parenting styles among mothers of pre‐ and adolescent children. On the basis of Levinson et al. (2022), we hypothesized that mothers with greater current depressive symptoms and blunted FN in response to their own monetary losses would report the use of more authoritarian styles with their children. We expected this finding because an authoritarian parenting style is defined by rigid beliefs and values about parenting and lack of responsiveness to children's needs for independence (Goodman 2020; Goodman and Gotlib 1999). This creates a parent–child relationship that is fraught by conflict and rigid and controlling parent–child communication styles. As such, the core features of authoritarian parenting style might be underpinned by a blunted FN profile in response to monetary loss feedback, which is proposed to reflect deficits in cognitive control, behavioral rigidity, and inflexibility and is associated with reduced adjustment to negative environmental cues (Cavanagh and Frank 2014). A blunted FN could additively increase risk for a more authoritarian parenting style among depressed mothers who are already at heightened vulnerability for parenting challenges. Given documented associations between RewP and reduced positive affect, social withdrawal, and depression (Kujawa and Burkhouse 2017; Kujawa et al. 2019), we expected that blunted RewP would be associated with lower warmth as blunted responsiveness to reward feedback might result in being less responsive to positive cues during parent–child interactions, and it is likely that these effects could be particularly pronounced among mothers with greater depressive symptom severity. Given evidence that even mothers with subclinical depressive symptoms exhibit nonoptimal parenting (Conners‐Burrow et al. 2014), we measured depressive symptoms dimensionally in a sample of community mothers with and without diagnostic histories of depression to understand at what levels of depressive symptoms mothers might increasingly adopt a problematic parenting style and if neural risk markers of RewP and FN moderate these associations.

### 1.3 Present Study

To empirically evaluate our questions, the study drew upon data from two larger intergenerational studies of the transmission of maternal depression to offspring. The goal was to recruit two groups of mothers with biological pre‐ and adolescent offspring from the community, a group with lifetime histories of MDDs and a separate group of mothers who were lifetime free of psychiatric illness. Departing from prior studies in this area (Levinson et al. 2017, 2022), we recruited a sample of mothers from diverse racial, socioeconomic, and ethnic backgrounds to increase the generalizability of findings. We hypothesized that maternal RewP would moderate the association between maternal depression (current symptoms and diagnostic history) and warm parenting styles, such that the association between maternal depression and reduced maternal warmth would only be observed among mothers exhibiting an attenuated RewP, indicating reduced reward responsiveness. In addition, we expected that maternal FN would moderate the relation between maternal depression (current symptoms and diagnostic history) and authoritarian parenting styles, such that the relation between increased authoritarian styles and maternal depression would only be observed among mothers exhibiting reduced FN (i.e., insensitivity to loss feedback).

## 2 Materials and Methods

### 2.1 Participants

Participants were 136 mothers (*M*_age_ = 42.18 years, SD = 6.57, range = 28–56 years) and their biological offspring (*M*_age_ = 12.41 years, SD = 2.15, range = 9–16 years old) who were participating in two studies on the intergenerational transmission of maternal depression. To be included in the present study, participants had to have complete data on all the variables in the analyses. Recruitment strategies, study procedures, and research personnel were identical across the two studies. Mothers were recruited in a large Midwestern metropolitan city using flyers, community events, and internet postings (e.g., Facebook). Two groups of mothers along with their offspring were recruited for the study, including an MDD group, which was comprised of mothers with a lifetime diagnosis of MDD (current and/or past, *n* = 81), and a psychiatrically healthy control (HC) comparison group, which consisted of mothers who could not meet diagnostic criteria for any current or past psychiatric disorder during their lifetime (*n* = 55). Mothers in the MDD group who had histories of substance/alcohol dependence within the past 6 months or histories of bipolar disorder, schizophrenia, intellectual disability, serious medical conditions, pervasive developmental disorders, and current active suicidal ideation were not eligible to participate in the study.

Mothers identified as White (61%), African American (15.4%), Asian (9.6%), other or unknown race (8.8%), multiracial (4.4%), and American Indian or Alaskan Native (0.7%). In terms of ethnicity, 27.2% of mothers identified as Latino/Latinx. Median family income was $95,000–$100,000 (range = $0 to more than $115,000), with 18.4% of the sample with income levels equal to or below the poverty line based on calculated income‐to‐needs ratios (*M* = 3.11, SD = 1.49, range = 0–7). The University of Illinois Chicago Institutional Review Board approved study procedures prior to data collection. Informed consent was obtained from mothers after study procedures were explained to them.

### 2.2 Measures

#### 2.2.1 Diagnostic Interviews

Research coordinators under the supervision of trained clinical psychologists administered the Structured Clinical Interview for DSM‐5 (SCID‐5; First 2014) to determine group assignment and history of current and past DSM‐5 diagnoses. To be assigned to the HC group, the mother could not have a current or past psychiatric disorder. For the MDD group, 81 mothers had a lifetime history of an MDD. Sixty‐three of the 81 mothers in the sample met criteria for recurrent MDD, and 12 met criteria for current MDD. Of the 81 mothers, 72 mothers’ MDD episodes occurred during their child's lifetime. Some of the mothers in the MDD group also met DSM‐5 criteria for other lifetime and current psychiatric disorders. A portion of mothers in the MDD group had lifetime histories of agoraphobia (*n *= 4), panic disorder (PD; *n* = 8), social anxiety disorder (SAD; *n *= 17), specific phobia (SP; *n *= 7), generalized anxiety disorder (GAD; *n *= 16), other specified anxiety disorder (OAD; *n* = 1), obsessive–compulsive disorder (OCD; *n* = 4), and post‐traumatic stress disorder (PTSD; *n* = 23). Twenty‐nine mothers met current diagnostic criteria for one or more of the following disorders: agoraphobia (*n* = 4), PD (*n *= 3), SAD (*n *= 17), SP (*n* = 7), GAD (*n *= 9), PTSD (*n* = 5), and OCD (*n* = 3).

#### 2.2.2 Depressive Symptoms

Mothers completed the Beck Depression Inventory‐Second Edition (BDI‐II; Beck et al. 1996), which is a measure of adult depressive symptoms within the past 2 weeks. Among the mothers in the sample, 11% reported depressive symptoms in the moderate‐to‐severe range. The total score was used in the analyses and demonstrated excellent internal consistency (*α* = 0.92).

#### 2.2.3 Parenting Styles

Mothers completed the Parental Bonding Instrument (PBI) (Kendler 1996; Parker et al. 1979) that assesses mother's attitudes and behaviors toward the child participant who was participating with them in the study. Maternal warmth subscale assesses engagement and responsiveness to children's emotional states and interests. Items include “I speak to my child in a warm and friendly voice” and “Appear to understand my child's problems and worries.” The authoritarianism subscale measures parental restrictiveness of their children's behavior. Items include “Like my child to make his/her own decisions.” and “Let my child dress in any way s/he pleases.” Items for both subscales are scored on a 4‐point Likert scale (0 = Very Like, 1 = Moderately Like, 2 = Moderately Unlike, 3 = Very Unlike), and some items are reverse scored. On both subscales, higher scores indicate higher levels of the parenting style (e.g., greater warmth and greater authoritarianism). Warmth (*α* = 0.81) and authoritarianism (*α* = 0.67) subscales were significantly negatively correlated (*r* = −0.18, *p* < 0.05).

#### 2.2.4 Doors Reward Task

Mothers completed a reward processing guessing game (i.e., doors monetary incentive task) that was administered on a computer using Presentation software. During the task, participants were shown an image of two identical doors—one on the left and the other on the right. The experimenter instructed the participants to select a door using the left or right mouse button. Before the task began, experimenters told participants that receiving feedback in the form of a green arrow indicated that they chose the right door and won $0.50, whereas seeing a red arrow indicated that they chose the wrong door and lost $0.25. After the selection was made, a fixation cross appeared on the screen for 1000 ms, followed by either a green arrow pointing up, indicating that the participant guessed correctly and won $0.50, or a red arrow pointing down, indicating that the participant guessed incorrectly and lost $0.25. These amounts were chosen to give gains and losses equivalent subjective values (Tversky and Kahneman 1992). After the arrow, a fixation mark is presented for 1500 ms, followed by a screen reading “click for the next round.” Once the participant responded, a new trial would begin. All participants completed 20 gain trials and 20 loss trials. Images were displayed on a black background.

### 2.3 EEG Data Acquisition and Processing

**FIGURE 1:**
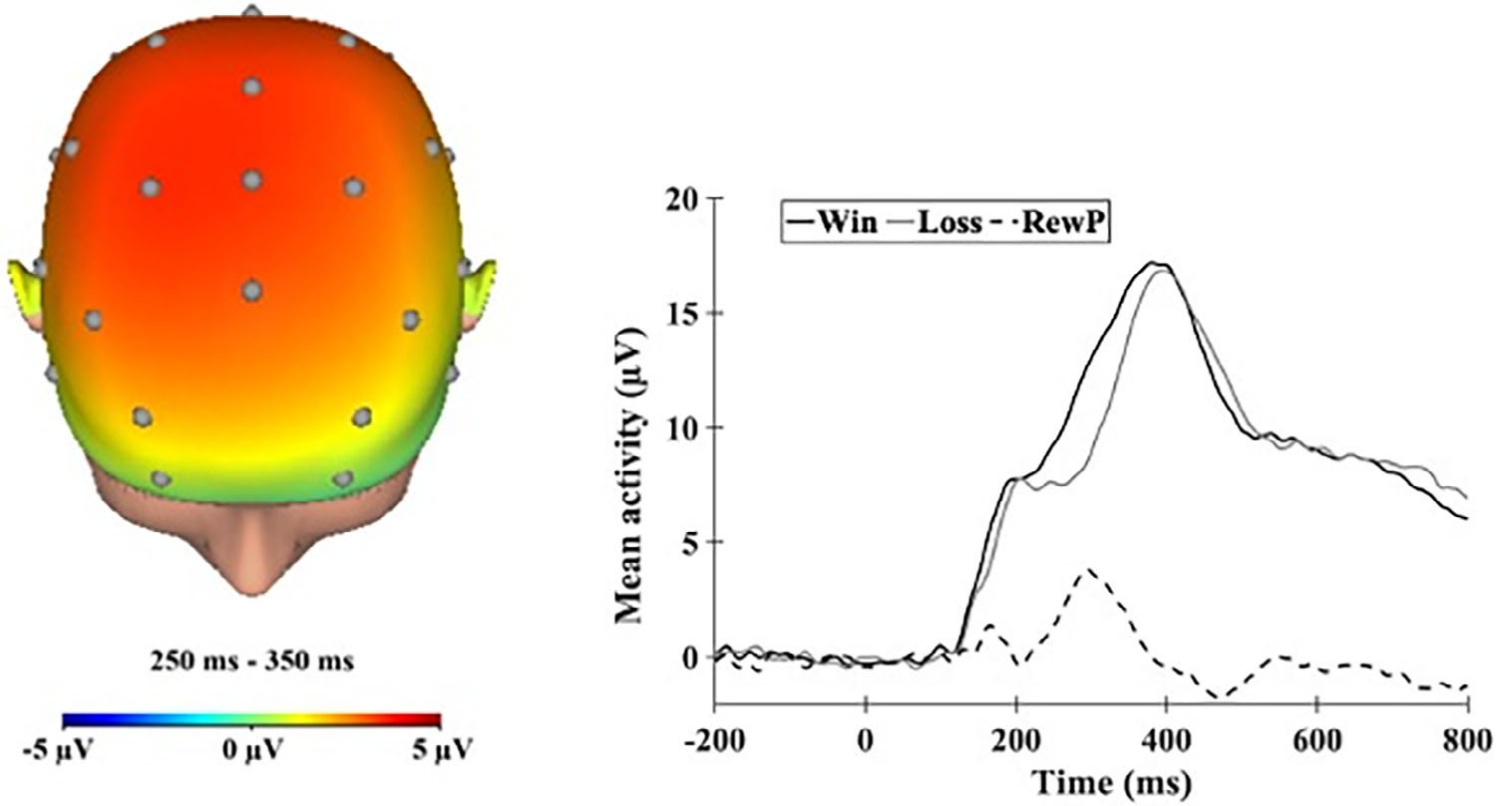
The topographic map of neural activity (win–loss) and response‐locked event‐related potential waveforms at an average of Fz, FCz, and Cz are plotted. The raw waveforms for win, loss, and In the graph, the waveforms depict the raw values for win and loss and residualized RewP score are shown in the graph. RewP, reward positivity.

Continuous EEG was recorded during the task using the ActiveTwo BioSemi system (BioSemi, Amsterdam, the Netherlands). This system uses a 32‐standard‐electrode cap with FCz and Iz added, totaling 34 electrodes. One electrode was placed on each mastoid. The EEG signal was pre‐amplified at the electrode to improve the signal‐to‐noise ratio. To account for ocular artifacts that result from eye blinks and other eye movements, electrooculogram (EOG) signals were recorded from two electrodes placed 1 cm above and below the right eye to measure vertical eye blinks and movements, and two electrodes were placed 1 cm beyond the outer edge of each eye to measure horizontal eye blinks and movements. The data were digitized at 24‐bit resolution with a Least Significant Bit value of 31.25 nV and a sampling rate of 1024 Hz. The voltage from each active electrode was referenced online with respect to a common‐mode sense active electrode. Offline analyses were performed using BrainVision Analyzer 2 software (Brain Products, Gilching, Germany). Data were re‐referenced to the average of the two mastoids and high‐pass (0.1 Hz) and low‐pass (30 Hz) filtered. Standard eyeblink and ocular corrections were performed (Gratton et al. 1983), and semiautomated artifact rejection procedures removed artifacts with the following criteria: voltage step of more than 50 µV between sample points, a voltage difference of 300 µV within a trial, and a maximum voltage difference of less than 0.5 µV within 100 ms intervals. Additional artifacts were removed using visual inspection. Data were baseline corrected using the 200 ms interval prior to feedback. ERPs were averaged across gain and loss trials, and the FN and RewP were both scored as the mean amplitude 250–350 ms following feedback at a pooling of FCz, Fz, and Cz sites where differences between gain and loss were maximal. Given the ΔRewP (gain‐loss) and ΔFN (loss‐gain) are mirror opposites of each other, Figure 1 only depicts the scalp topography and waveform for the residualized RewP score. FN was quantified by regressing the loss on the win score to isolate the variance related to mother's responsiveness to loss feedback. Similarly, RewP was calculated by regressing the win score on the loss score to isolate the variance related to mother's responsiveness to reward feedback. The unstandardized residuals for win and loss were saved and used in the analyses. The average number of included trials following artifact rejection across participants was 18.30 (SD = 2.74) for win and 18.29 (SD = 2.81) for loss trials. MDD and HC groups did not differ in the average number of win (*t* (134) = 0.52, *p* = 0.61) or loss (*t* (134) = 0.18, *p* = 0.86) trials. There was a significant negative correlation between residual FN and RewP scores (*r* = −0.80, *p* < 0.001).

### 2.4 Data Analytic Plan

For our main analyses, we conducted a series of two‐way interaction analyses using the SPSS PROCESS Macro Version 4.2 (Hayes 2022) in SPSS Version 29 software (IBM Corp., Armonk, N.Y., USA). In each model, we examined maternal current depressive symptoms as the predictor variable and FN and RewP as the moderating variables. Warmth and authoritarian parenting styles were examined as dependent variables in our regression analyses. We also conducted supplemental regression analyses where we included the categorical group variable (MDD vs. HC) in the model rather than current depressive symptoms to determine whether effects differed depending on whether depression was measured categorically as opposed to dimensionally. Because we observed positive bivariate correlations between child gender and age and our study variables, we included them as covariates in all the analyses. Additionally, we covaried for the potential influence of family income‐to‐needs given that there was significant variability in family income in our sample, and prior work has found that mothers from lower economic backgrounds are at higher risk for problematic parenting (Callaghan et al. 2024; Sturge‐Apple et al. 2017). We performed simple slope analyses on significant two‐way interactions between maternal ERPs and maternal depressive symptoms in relation to the two parenting styles. For these analyses, maternal RewP and FN were set as moderators, and their slopes were plotted at −1/+1 SD from their centered means. Maternal current depressive symptoms were plotted at −/+1 SD from the centered mean.

## 3 Results

Table 1 shows demographic and clinical characteristics, and whether MDD and HC groups differed from each other across these variables. There were significant differences in offspring age and gender distribution across the HC and MDD groups, such that youth of HC mothers were on average older, and there were more offspring that identified as females in the MDD group compared to the HC group. As to be expected, the MDD group reported greater current depressive symptoms compared to the HC group. Table 2 displays the means, standard deviations, and correlations among the primary variable in the analyses. Current maternal depressive symptoms and group assignment were moderately correlated (*r* = 0.42, *p* < 0.001).

**TABLE 1:** Demographic and clinical characteristics of healthy controls (HC) and major depressive disorder (MDD) mothers.

	HC (*n* = 55)		MDD (*n* = 81)		Overall (*n* = 136)			
	*M*	SD	*M*	SD	*M*	SD	*F*	*p* value
Child age	13.09	0.21	11.95	2.10	12.41	2.15	9.80	0.01
Income‐to‐needs	3.16	1.42	3.08	1.54	3.11	1.49	0.10	0.77
BDI‐II total score	3.50	4.82	10.85	9.41	7.88	8.65	35.76	0.01
	*N*	%	*N*	%	*N*	%	*χ* ^2^	*p* value
*Child gender*							5.32	0.02
Female	48	45.7	57	54.3	105	77.3		
*Maternal ethnicity*							0.0	0.99
Latino/Latinx	15	11.0	22	16.2	37	27.2		
*Maternal race*							3.01	0.70
White	36	26.5	47	34.6	83	61.0		
Black	7	5.1	14	10.3	21	15.4		
Am. Ind. or Ala. Nat.	0	0	1	0.7	1	0.7		
Asian	6	4.4	7	5.1	13	9.6		
More than 1 race	1	0.7	5	3.7	6	4.4		
Unknown or other	5	3.7	7	5.1	12	8.8		
*Comorbid disorders (current/lifetime combined)*								
Panic	—	—	8	5.9				
Social anxiety	—	—	17	12.6				
GAD	—	—	16	11.9				
Specific phobia	—	—	7	5.2				
Agoraphobia			4	3.0				
Other anxiety	—	—	1	0.7				
OCD	—	—	4	3.0				
PTSD	—	—	23	17.0				

Abbreviations: Am. Ind. or Ala. Nat., American Indian or Alaskan Native; BDI‐II, Beck Depression Inventory‐Second Edition; GAD, generalized anxiety disorder; OCD, obsessive‐compulsive disorder; PTSD, post‐traumatic stress disorder.

**TABLE 2:** Intercorrelations among variables.

Variable	1	2	3	4	5	6	7	8
1. Child age	—							
2. Female	0.28**	—						
3. Income‐to‐needs	0.09	0.16	—					
4. MDD group	−0.26**	−0.20*	−0.03	—				
5. BDI‐II total score	−0.09	−0.18*	−0.10	0.42**	—			
6. Res. RewP	−0.12	−0.03	−0.15	0.04	−0.08	—		
7. Res. FN	0.05	0.04	0.12	−0.01	0.11	−0.80**	—	
8. PBI warmth	−0.08	−0.02	0.03	−0.15	−0.33**	−0.15	0.15	—
9. PBI authoritarianism	0.01	0.10	−0.12	−0.10	0.07	0.16	−0.10	−0.18*

Abbreviations: BDI‐II, Beck Depression Inventory‐Second Edition; FN, feedback negativity; MDD, major depressive disorder; PBI, Parental Bonding Instrument; Res., residual; RewP, reward positivity.

*p < 0.05.

**p < 0.01.

### 3.1 Maternal RewP and Parenting

We first explored the main and two‐way interaction effects between maternal current depressive symptoms and RewP in association with maternal warmth and authoritarian parenting styles, respectively (see Table 3). For maternal warmth, we observed a direct effect of maternal current depressive symptoms. Mothers with greater current depressive symptoms reported being less warm with their offspring (*b* = −0.11, SE = 0.03, *t* = −3.99, *p* < 0.001, 95% CI −0.16, −0.05). The main effect of RewP (*b *= 0.09, SE = 0.06, *t* = 1.45, *p* = 0.15, 95% CI −0.03, 0.20) and the two‐way interaction between maternal current depressive symptoms and RewP in association with warmth were not significant (*b* = 0.00, SE = 0.01, *t* = 0.47, *p* = 0.64, 95% CI −0.01, 0.02). In the next model, we examined authoritarian parenting styles as the dependent variable. Neither the direct effect of RewP nor the two‐way interaction effect between maternal current depressive symptoms and RewP in association with authoritarian parenting style was significant (see Table 3 for full results).

**TABLE 3:** Depressive symptoms and reward positivity (RewP) interactions with parenting styles.

*Warmth*						
	*b*	SE	*t*	*p*	95% LL CI	95% UL CI
Child age	−0.10	0.11	−0.92	0.36	−0.32	0.12
Female	−0.36	0.57	−0.64	0.52	−1.50	0.77
Income‐to‐needs	0.05	0.16	0.30	0.77	−0.27	0.36
BDI‐II total	−0.11	0.03	−3.99	0.00	−0.16	−0.05
Res. RewP	0.09	0.06	1.45	0.15	−0.03	0.20
BDI ‐II total × Res. RewP	0.00	0.01	0.47	0.64	−0.01	0.02
*Authoritarianism*						
Child age	−0.03	0.10	−0.23	0.74	−0.20	0.14
Female	0.69	0.45	1.56	0.12	−0.19	1.58
Income‐to‐needs	−0.19	0.12	−1.53	0.13	−0.43	0.05
BDI‐II total	0.02	0.02	0.93	0.35	−0.02	0.06
Res. RewP	−0.06	0.05	−1.34	0.18	−0.15	0.03
BDI‐II total × Res. RewP	−0.01	0.01	−1.58	0.12	−0.02	0.00

Abbreviations: BDI‐II, Beck Depression Inventory‐Second Edition; Res., residual; RewP, reward positivity.

We also conducted identical analyses with MDD group rather than maternal current depressive symptoms in the models, and similar findings were observed. The only significant effect across the analyses was that mothers with histories of MDD (current/lifetime) reported being less warm with their children (*b* = −1.08, SE = 0.50, *t* = −2.15, *p* = 0.03, 95% CI −2.07, −0.09).

### 3.2 Maternal FN and Parenting Styles

In the next set of regression analyses, we examined the main and two‐way interaction effects between maternal current depressive symptoms and FN in relation to the two parenting styles. Like the RewP and warm parenting style results, we only observed a significant main effect of maternal current depressive symptoms in association with maternal warmth, such that greater current depressive symptom severity was associated with lower maternal warmth (*b* = −0.11, SE = 0.03, *t* = −3.93, *p* < 0.001, 95% CI −0.16, −0.05; see Table 4 for results). Although the overall model did not reach significance, MDD group was also associated with mothers reporting less warmth in their parenting style with their offspring compared to HC mothers (*b* = 1.05, SE = 0.50, *t* = −2.10, *p *= 0.04, 95% CI −2.05, −0.06; Table 4).

**TABLE 4:** Depressive symptoms and feedback negativity (FN) interactions with parenting styles.

*Warmth*						
	*b*	SE	*t*	*p*	95% LL CI	95% UL CI
Child age	−0.12	0.11	−1.05	0.29	−0.33	0.10
Female	−0.34	0.57	−0.59	0.56	−1.47	0.80
Income‐to‐needs	0.04	0.16	0.28	0.78	−0.27	0.35
BDI‐II total	−0.11	0.03	−3.93	0.00	−0.16	−0.05
Res. FN	−0.09	0.07	−1.28	0.20	−0.23	0.05
BDI‐II total × Res. FN	−0.00	0.01	−0.09	0.93	−0.02	0.02
*Authoritarianism*						
Child age	0.00	0.08	0.02	0.98	−0.16	0.17
Female	0.62	0.44	1.43	0.16	−0.24	1.47
Income‐to‐needs	−0.20	0.12	−1.70	0.09	−0.43	0.03
BDI‐II total	0.01	0.02	0.71	0.48	−0.03	0.05
Res. FN	0.11	0.05	2.04	0.04	0.00	0.21
BDI‐II Total × Res. FN	0.02	0.01	3.00	0.00	0.01	0.03

Abbreviations: BDI‐II, Beck Depression Inventory‐Second Edition; Res., residual.

**FIGURE 2:**
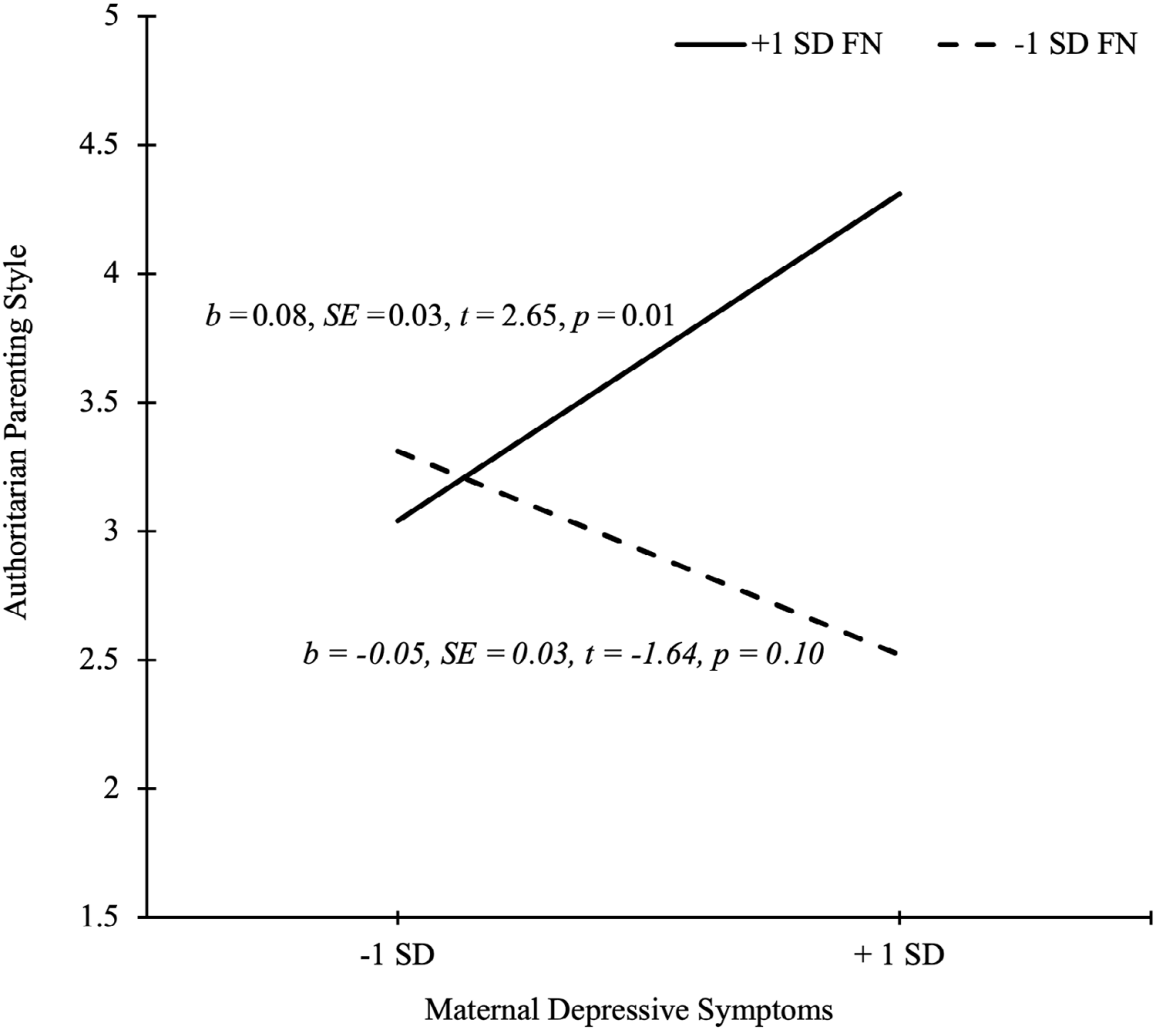
Plot of the significant interaction between maternal depressive symptoms and feedback negativity (FN) on authoritarian parenting style. Values on the *x*‐axis are centered maternal depression scores on the BDI‐II. Solid line denotes the significant slope for mothers with +1 SD from the centered mean on FN, whereas the dashed line represents the non‐significant slope for mothers with −1 SD from the centered mean on FN. FN is a negative event‐related potential component. More negative amplitude values (−1 SD) reflect more neural activity to loss feedback, whereas less negative amplitude values (+1 SD) indicate a blunted response to loss feedback.

When we examined authoritarian parenting style in the model as the dependent variable, we did not detect a main effect of maternal current depressive symptoms (*b* = 0.01, SE = 0.02, *t *= 0.71, *p* = 0.48, 95% CI −0.03, 0.05), but there was a main effect of maternal FN in relation to maternal authoritarian parenting style (*b* = 0.11, SE = 0.05, *t* = −2.04, *p* = 0.04, 95% CI 0.00, 0.21). However, the direct effect of maternal FN on authoritarian parenting style was qualified by a significant interaction with maternal current depressive symptoms (*b *= 0.02, SE = 0.01, *t* = 3.00, *p* = 0.03, 95% CI 0.01, 0.03; see Table 4), which we followed up with a simple slope analysis. For mothers with more positive FN amplitudes, indicating a blunted sensitivity to loss feedback, greater current depressive symptoms were associated with more authoritarian parenting styles (*b* = 0.08, SE = 0.03, *t* = 2.65, *p* = 0.01, 95% CI 0.02, 0.13). For mothers with more negative FN amplitudes (e.g., enhanced sensitivity to loss feedback), maternal current depressive symptoms were not associated with authoritarian parenting styles (*b* = −0.05, SE = 0.03, *t* = −1.64, *p* = 0.10, 95% CI −0.11, 0.01; see Figure 2). As in the other analyses, we tested whether similar effects were observed with diagnostic (MDD vs. HC) group rather than maternal current depressive symptoms in the model. None of the effects reached statistical significance (*p*s > 0.05).

## 4 Discussion

Maternal depression is a major public health concern, impacting 10% of children per year (Ertel et al. 2011). Additionally, mothers who experience depression during the postpartum period are more likely to experience depressive symptoms at later stages of parenthood (Trussell et al. 2018). As such, they continue to be a greater risk for mental health challenges beyond infancy, which has significant negative consequences for their offspring at all stages of their development (Gutierrez‐Galve et al. 2019; Hammen and Brennan 2003). Crucially, some mothers with depression struggle more than others in their parenting role, with increasing evidence suggesting that alterations in neural processes that support parenting behaviors and styles in conjunction with emotional and social stressors may contribute to nonoptimal parenting (Barrett and Fleming 2011; Callaghan et al. 2024; Hajal and Loo 2021). However, we still know very little about how neural processes implicated in affective and motivational behavior and their potential interaction with depression contribute to parenting outcomes, particularly among at‐risk mothers with older children. The present study sought to address this gap by evaluating the interplay between maternal depression and neurophysiological markers of reward and loss responsiveness in relation to two core parenting dimensions, warmth and authoritarianism. Contrary to expectations, we did not find an interactive effect between maternal current depressive symptoms and RewP in relation to maternal ratings of warmth. We did detect a significant main effect of maternal current depressive symptoms in association with reduced warmth. This finding is largely consistent with the broader literature on maternal depression and reduced warmth during parent–child interactions (Foster et al. 2008; Lovejoy et al. 2000; Trussell et al. 2018; Turney 2011). Our finding that there was not a main effect of RewP on maternal warmth is consistent with prior studies demonstrating null associations between observational RewP and active RewP and parent‐reported authoritative parenting style, a parenting style defined as being highly responsive to children's emotional, physical, and psychological needs (Levinson et al. 2017, 2022). One explanation is that other ERPs could have stronger associations with sensitive parenting compared to RewP elicited during vicarious and active reward tasks (Hajal and Loo 2021). For example, a few studies have found that maternal N170, an index of facial emotion processing and late positive potential, measuring neural response to emotionally provocative stimuli, are associated with maternal sensitivity among mothers with young children (Dudek and Haley 2020; Kuzava et al. 2019; Rutherford et al. 2018). Future empirical investigations should consider evaluating ERPs that index different cognitive and affective processes and their putative role in supporting maternal sensitivity within the context of maternal depression and across later stages of their child's development.

As predicted, we detected a unique interactive effect between maternal current depressive symptoms and FN in association with mother‐reported authoritarian parenting style. Mothers endorsing greater levels of current depressive symptoms and who exhibited a blunted FN (e.g., reduced responsiveness to monetary loss feedback) reported using a more authoritarian parenting style (e.g., being more behaviorally and psychologically controlling with their children). Neuroimaging and ERP studies that have examined neural responses to loss feedback indicate that the FN is generated by the ACC (Cohen and Ranganath 2007; Luu et al. 2003, 2004), which is a brain region that supports making appropriate behavioral adjustments to achieve desired outcomes after receiving negative performance cues. Reduced activation in the ACC is associated with cognitively inflexibility and rigid behavior patterns. The OFC is another region that is activated in response to punishment (Wrase et al. 2007), and clinical samples who exhibit patterns of under activation in response to punishment have also been found to display insensitivity to the social consequences of their own behavioral choices (Anderson et al. 2013; Bechara 2004; Bechara et al. 2000). Drawing upon this neuroimaging work and the proposed functional significance of the FN for motivated behavior, one tentative interpretation of our FN and authoritarian parenting style finding is that reduced neural responsiveness to negative feedback could increase risk for high levels of behavioral control, more rigid beliefs about compliance with parental authority, and difficulties with parent–child conflict resolution, which represent core characteristics of an authoritarian parenting style.

Notably, the current findings suggest that the effect of maternal FN on authoritarian parenting style might increase as maternal depressive symptoms become more severe. Conversely, the association between maternal current depressive symptoms and authoritarian parenting style was not significant for mothers who exhibited a more heightened FN, which could suggest that greater reactivity to loss or negative feedback buffers mothers from being more authoritarian with their children even as their depressive symptoms increase. This could be because their neural profiles help them to maintain a flexible response to their children's changing developmental needs regardless of whether they are experiencing depressive symptoms. Another tentative hypothesis and interpretation of the findings is that mothers with heightened FN might be able to incorporate feedback from their children about fairness of behavioral expectations or pick up on negative cues from their children indicating that their parenting style is causing conflict or damage to their relationship with their child. Given the cross‐sectional design and the limited measurement battery, these interpretations are highly speculative and should be interpreted with caution until replication. Specifically, the monetary task we used did not require mothers to adjust their behavior based on reward or punishment feedback. Future work should consider using different monetary incentive tasks that prompt participants to modify behavior based on reinforcement cues to evaluate the validity of these hypotheses and tentative interpretations. Lastly, even though an enhanced FN might be protective against authoritarian parenting styles among mothers with depressive symptoms, it might increase risk for other problematic parenting styles and behaviors that we did not assess in the current study.

Given the high‐ and low‐risk design of the sample, we also evaluated the potential interplay between MDD diagnostic history and FN in relation to authoritarian parenting styles by including the diagnostic grouping variable rather than current depressive symptoms in the models. Interestingly, the interaction between MDD history and FN did not reach statistical significance for authoritarian parenting, suggesting that the interactive effect is driven by a mother's current depressive symptoms. Importantly, relatively few mothers in the study met criteria for current MDD, and across the whole sample current symptoms fell in modest‐to‐moderate ranges of severity. In fact, inspection of the simple slope crossover‐point suggests that maternal FN and authoritarian parenting style became correlated below the centered mean of depressive symptoms, which could potentially suggest that effects might begin at what are likely subclinical levels of depression and then steadily increase as depressive symptoms become more severe. This is unsurprising given the substantial body of research that has demonstrated that subclinical mental health symptoms can lead to significant psychosocial impairments (Angold et al. 1999; Conners‐Burrow et al. 2014; Merikangas et al. 2010). Considering this, it will be important for providers in primary care settings to not solely rely on clinical cutoffs on depression screening forms for identifying which mothers could benefit from parenting programs and caregiving support.

Another key issue to consider is that most primary care providers only administer depression screens to postpartum mothers at birth to 12‐month infant well visits (Trussell et al. 2018). This stands in contrast to a wealth of empirical knowledge that demonstrates that maternal depression can have a negative impact on parenting well beyond infancy and is associated with detrimental health, social, educational, and emotional outcomes in older children and adolescents (Foster et al. 2008; Goodman and Gotlib 1999; Gutierrez‐Galve et al. 2019). Given the high prevalence of depression among mothers and the devastating impact on youth across all stages of their development (Ertel et al. 2011), public health agencies should advocate for implementing maternal depression screenings during all pediatric wellness visits.

### 4.1 Limitations and Future Directions

Although the findings provide some novel insights into the role of neurophysiological indicators of motivational processes in maternal parenting styles, particularly for mothers with depressive symptoms, the study had some limitations. First, the study design was cross‐sectional, limiting our ability to examine longitudinal associations between maternal depression and parenting styles and testing whether RewP and FN patterns reflect stable or transient profiles among mothers. Another limitation of the current study is that the MDD group was diagnostically heterogenous, including mothers with lifetime histories of single and recurrent MDD and those with current MDD. A significant portion of mothers met criteria for recurrent MDD. However, we did not collect information on the total number of episodes mothers with recurrent MDD had experienced in their lifetime. Additionally, the sample size was not large enough to conduct fine‐grained group comparisons to elucidate whether findings differed across these MDD profiles; future studies would benefit from testing differences across these groups and evaluating how chronicity and severity of MDD might interact with neural mechanisms implicated in supporting different parenting practices and behaviors.

To increase precision in multilevel mechanistic models of maternal depression and offspring psychopathology risk, future investigations need to evaluate potential bidirectional effects between child characteristics (e.g., negative emotionality, mental health symptoms, and gender) and maternal neurobiological mechanisms on parenting. Interactional patterns between parents and their children can reciprocally impact parenting across development. One potential future direction could be to delineate how neuroaffective risk markers among parents and their offspring reciprocally contribute to increased parent–child conflict, especially as adolescent blunted RewP in response to social rewards and experiences of higher maternal conflict is associated with increased depression risk (Hill et al. 2023).

With respect to other methodological and design limitations, we only used mother's self‐report of their parenting styles. Research has supported the validity of the PBI for measuring actual characteristics of parenting (Parker 1983). Despite this, future work could consider incorporating observational ratings of parenting during parent–adolescent interactions, such as laboratory‐based conflict discussion tasks, and adolescent reports of their parent's parenting styles to understand if the use of adolescent report and observational measures of parenting produce similar findings. Lastly, the study only included mothers, which limits our understanding of how paternal neural responses to motivationally significant stimuli might interact with their mental health symptoms in relation to their parenting styles. Depressed fathers are significantly underrepresented in intergenerational transmission of depression research despite its prevalence—some recent estimates suggest that 8% of postpartum fathers experience depression (Cameron et al. 2020). Although rates of depression among fathers with older children are relatively unknown, meta‐analyses have documented the negative impact of paternal depression on parenting and child and adolescent outcomes (Cheung and Theule 2019; Goodman 2004; Gutierrez‐Galve et al. 2019; Shafer et al. 2017). Specific parenting practices typically displayed by fathers also play a unique role in shaping children's socioemotional development (Clinchard et al. 2024; Paquette 2004; Volling and Cabrera 2019). Thus, research that elucidates how paternal neuroaffective markers might also moderate associations between depression and parenting is crucial for supporting children's well‐being within the entire family system.

## 5 Conclusion

In sum, to our knowledge, this is the first study to evaluate whether ERP markers of reward and loss feedback act as moderating processes that account for why some depressed mothers with pre‐ and adolescent‐age children are at greater risk for parenting challenges. Findings suggest that mothers with neural profiles indicative of blunted responsiveness to negative cues, such as losing rewards, might be at particular risk for adopting an authoritarian parenting style as their depressive symptoms increase. Given the public health burden of maternal depression and its devastating developmental consequences for their offspring, it is crucial to identify malleable processes, across levels of influence, that can be modified by parenting interventions. Interventions that are tailored to parental characteristics and depart from the traditional treatment fits all approach have the potential to produce more enduring improvements in positive parenting styles, parent–child relationship quality, and offspring well‐being.

## Ethics Statement

The University of Illinois Chicago Institutional Review Board approved all study procedures.

## Conflicts of Interest

The authors declare no conflicts of interest.

## Data Availability

The data that support the findings of this study are available from the corresponding author upon reasonable request.

## References

[dev70083-bib-0001] Anderson, S. W. , A. Bechara , H. Damasio , D. Tranel , and A. R. Damasio . 2013. “Impairment of Social and Moral Behavior Related to Early Damage in Human Prefrontal Cortex.” In Social Neuroscience. Psychology Press. 10.1038/14833 10526345

[dev70083-bib-0002] Angold, A. , E. J. Costello , E. M. Z. Farmer , B. J. Burns , and A. Erkanli . 1999. “Impaired but Undiagnosed.” Journal of the American Academy of Child & Adolescent Psychiatry 38: 129–137. 9951211 10.1097/00004583-199902000-00011

[dev70083-bib-0003] Band, G. P. , H. van Steenbergen , K. R. Ridderinkhof , M. Falkenstein , and B. Hommel . 2009. “Action‐Effect Negativity: Irrelevant Action Effects Are Monitored Like Relevant Feedback.” Biological Psychology 82: 211–218. 19665516 10.1016/j.biopsycho.2009.06.011

[dev70083-bib-0004] Barrett, J. , and A. S. Fleming . 2011. “Annual Research Review: All Mothers Are Not Created Equal: Neural and Psychobiological Perspectives on Mothering and the Importance of Individual Differences: Neurobiological Perspectives on Mothering.” Journal of Child Psychology and Psychiatry 52: 368–397. 20925656 10.1111/j.1469-7610.2010.02306.x

[dev70083-bib-0005] Baumrind, D. 1971. “Current Patterns of Parental Authority.” Developmental Psychology 4: 1–103.

[dev70083-bib-0006] Bechara, A. 2004. “The Role of Emotion in Decision‐Making: Evidence From Neurological Patients With Orbitofrontal Damage.” Brain and Cognition 55: 30–40. 15134841 10.1016/j.bandc.2003.04.001

[dev70083-bib-0007] Bechara, A. , H. Damasio , and A. R. Damasio . 2000. “Emotion, Decision Making and the Orbitofrontal Cortex.” Cerebral Cortex 10: 295–307. 10731224 10.1093/cercor/10.3.295

[dev70083-bib-0008] Beck, A. , R. Steer , and G. Brown . 1996. Manual for the Beck Depression Inventory‐II (BDI‐II). Psychological Corporation.

[dev70083-bib-0009] Bernat, E. M. , L. D. Nelson , and A. R. Baskin‐Sommers . 2015. “Time‐Frequency Theta and Delta Measures Index Separable Components of Feedback Processing in a Gambling Task.” Psychophysiology 52: 626–637. 25581491 10.1111/psyp.12390 PMC4398588

[dev70083-bib-0010] Bernat, E. M. , L. D. Nelson , C. B. Holroyd , W. J. Gehring , and C. J. Patrick . 2008. “Separating Cognitive Processes With Principal Components Analysis of EEG Time‐Frequency Distributions.” Advanced Signal Processing Algorithms, Architectures, and Implementations XVIII 7074: 240–249.

[dev70083-bib-0011] Bernat, E. M. , L. D. Nelson , V. R. Steele , W. J. Gehring , and C. J. Patrick . 2011. “Externalizing Psychopathology and Gain–Loss Feedback in a Simulated Gambling Task: Dissociable Components of Brain Response Revealed by Time‐Frequency Analysis.” Journal of Abnormal Psychology 120: 352–364. 21319875 10.1037/a0022124 PMC3092030

[dev70083-bib-0012] Cairney, J. , M. Boyle , D. R. Offord , and Y. Racine . 2003. “Stress, Social Support and Depression in Single and Married Mothers.” Social Psychiatry and Psychiatric Epidemiology 38: 442–449. 12910340 10.1007/s00127-003-0661-0

[dev70083-bib-0013] Callaghan, B. L. , C. McCormack , P. Kim , and J. L. Pawluski . 2024. “Understanding the Maternal Brain in the Context of the Mental Load of Motherhood.” Nature Mental Health 2: 764–772.

[dev70083-bib-0014] Cameron, E. E. , K. M. Joyce , C. P. Delaquis , K. Reynolds , J. L. P. Protudjer , and L. E. Roos . 2020. “Maternal Psychological Distress & Mental Health Service Use During the COVID‐19 Pandemic.” Journal of Affective Disorders 276: 765–774. 32736186 10.1016/j.jad.2020.07.081 PMC7370903

[dev70083-bib-0015] Cavanagh, J. F. , and M. J. Frank . 2014. “Frontal Theta as a Mechanism for Cognitive Control.” Trends in Cognitive Sciences 18: 414–421. 24835663 10.1016/j.tics.2014.04.012 PMC4112145

[dev70083-bib-0016] Chad‐Friedman, E. , L. S. Jordan , S. Chad‐Friedman , et al. 2024. “Parent and Child Depressive Symptoms and Authoritarian Parenting: Reciprocal Relations From Early Childhood Through Adolescence.” Clinical Psychological Science 12: 403–420.

[dev70083-bib-0017] Cheung, K. , and J. Theule . 2019. “Paternal Depressive Symptoms and Parenting Behaviors: An Updated Meta‐Analysis.” Journal of Child and Family Studies 28: 613–626.

[dev70083-bib-0018] Clinchard, C. , K. Deater‐Deckard , B. Casas , and J. Kim‐Spoon . 2024. “Longitudinal Links From Attachment With Mothers and Fathers to Adolescent Substance Use: Internalizing and Externalizing Pathways.” Developmental Science 27, no. 6: e13539. 39031676 10.1111/desc.13539 PMC12517308

[dev70083-bib-0019] Cohen, M. X. , and C. Ranganath . 2007. “Reinforcement Learning Signals Predict Future Decisions.” Journal of Neuroscience 27: 371–378. 17215398 10.1523/JNEUROSCI.4421-06.2007 PMC6672075

[dev70083-bib-0020] Conners‐Burrow, N. A. , P. Bokony , L. Whiteside‐Mansell , et al. 2014. “Low‐Level Depressive Symptoms Reduce Maternal Support for Child Cognitive Development.” Journal of Pediatric Health Care: Official Publication of National Association of Pediatric Nurse Associates & Practitioners 28: 404–412. 24503001 10.1016/j.pedhc.2013.12.005

[dev70083-bib-0021] Darling, N. , and L. Steinberg . 2017. “Parenting Style as Context: An Integrative Model.” In Interpersonal Development. Routledge.

[dev70083-bib-0022] Deater‐Deckard, K. , and M. A. Bell . 2017. “Maternal Executive Function, Heart Rate, and EEG Alpha Reactivity Interact in the Prediction of Harsh Parenting.” Journal of Family Psychology 31: 41–50. 28165280 10.1037/fam0000286 PMC5302022

[dev70083-bib-0023] Deater‐Deckard, K. , N. Chen , Z. Wang , and M. A. Bell . 2012. “Socioeconomic Risk Moderates the Link Between Household Chaos and Maternal Executive Function.” Journal of Family Psychology 26: 391–399. 22563703 10.1037/a0028331 PMC3368074

[dev70083-bib-0024] Deater‐Deckard, K. , Z. Wang , N. Chen , and M. A. Bell . 2012. “Maternal Executive Function, Harsh Parenting, and Child Conduct Problems.” Journal of Child Psychology and Psychiatry 53: 1084–1091. 22764829 10.1111/j.1469-7610.2012.02582.x PMC3460037

[dev70083-bib-0025] Dougherty, L. R. , M. R. Tolep , V. C. Smith , and S. Rose . 2013. “Early Exposure to Parental Depression and Parenting: Associations With Young Offspring's Stress Physiology and Oppositional Behavior.” Journal of Abnormal Child Psychology 41: 1299–1310. 23722864 10.1007/s10802-013-9763-7

[dev70083-bib-0026] Dudek, J. , and D. W. Haley . 2020. “Attention Bias to Infant Faces in Pregnant Women Predicts Maternal Sensitivity.” Biological Psychology 153: 107890. 32335127 10.1016/j.biopsycho.2020.107890

[dev70083-bib-0027] Ertel, K. A. , J. W. Rich‐Edwards , and K. C. Koenen . 2011. “Maternal Depression in the United States: Nationally Representative Rates and Risks.” Journal of Women's Health 20, no. 11: 1609–1617. 10.1089/jwh.2010.2657 PMC3253390 21877915

[dev70083-bib-0028] First, M. B. 2014. “Structured Clinical Interview for the DSM (SCID).” In The Encyclopedia of Clinical Psychology. Wiley.

[dev70083-bib-0029] Forman, D. R. , M. W. O'HARA , S. Stuart , L. L. Gorman , K. E. Larsen , and K. C. Coy . 2007. “Effective Treatment for Postpartum Depression Is Not Sufficient to Improve the Developing Mother–Child Relationship.” Development and Psychopathology 19: 585–602. 17459185 10.1017/S0954579407070289

[dev70083-bib-0030] Foster, C. J. , J. Garber , and J. A. Durlak . 2008. “Current and Past Maternal Depression, Maternal Interaction Behaviors, and Children's Externalizing and Internalizing Symptoms.” Journal of Abnormal Child Psychology 36: 527–537. 18071896 10.1007/s10802-007-9197-1

[dev70083-bib-0031] Foti, D. , A. Weinberg , E. M. Bernat , and G. H. Proudfit . 2015. “Anterior Cingulate Activity to Monetary Loss and Basal Ganglia Activity to Monetary Gain Uniquely Contribute to the Feedback Negativity.” Clinical Neurophysiology 126: 1338–1347. 25454338 10.1016/j.clinph.2014.08.025 PMC4385748

[dev70083-bib-0032] Frye, A. A. , and J. Garber . 2005. “The Relations Among Maternal Depression, Maternal Criticism, and Adolescents? Externalizing and Internalizing Symptoms.” Journal of Abnormal Child Psychology 33: 1–11. 15759587 10.1007/s10802-005-0929-9

[dev70083-bib-0033] Gehring, W. J. , and A. R. Willoughby . 2002. “The Medial Frontal Cortex and the Rapid Processing of Monetary Gains and Losses.” Science 295: 2279–2282. 11910116 10.1126/science.1066893

[dev70083-bib-0034] Goodman, J. H. 2004. “Paternal Postpartum Depression, Its Relationship to Maternal Postpartum Depression, and Implications for Family Health.” Journal of Advanced Nursing 45: 26–35. 14675298 10.1046/j.1365-2648.2003.02857.x

[dev70083-bib-0035] Goodman, S. H. , and I. H. Gotlib eds. 2002. Children of Depressed Parents: Mechanisms of Risk and Implications for Treatment. American Psychological Association.

[dev70083-bib-0036] Goodman, S. H. 2020. “Intergenerational Transmission of Depression.” Annual Review of Clinical Psychology 16: 213–238. 10.1146/annurev-clinpsy-071519-113915 31961725

[dev70083-bib-0037] Goodman, S. H. , and I. H. Gotlib . 1999. “Risk for Psychopathology in the Children of Depressed Mothers: A Developmental Model for Understanding Mechanisms of Transmission.” Psychological Review 106: 458–490. 10467895 10.1037/0033-295x.106.3.458

[dev70083-bib-0038] Goodman, S. H. , M. H. Rouse , A. M. Connell , M. R. Broth , C. M. Hall , and D. Heyward . 2011. “Maternal Depression and Child Psychopathology: A Meta‐Analytic Review.” Clinical Child and Family Psychology Review 14: 1–27. 21052833 10.1007/s10567-010-0080-1

[dev70083-bib-0086] Goodman, S. H. , H. F. Simon , A. L. Shamblaw , and C. Y. Kim . 2020. “Parenting as a Mediator of Associations Between Depression in Mothers and Children's Functioning: a Systematic Review and Meta‐analysis.” Clinical Child and Family Psychology Review 23: 427–460. 32734498 10.1007/s10567-020-00322-4

[dev70083-bib-0039] Gottfried, J. A. , and R. J. Dolan . 2004. “Human Orbitofrontal Cortex Mediates Extinction Learning While Accessing Conditioned Representations of Value.” Nature Neuroscience 7: 1144–1152. 15361879 10.1038/nn1314

[dev70083-bib-0040] Gratton, G. , M. G. Coles , and E. Donchin . 1983. “A New Method for off‐Line Removal of Ocular Artifact.” Electroencephalography and Clinical Neurophysiology 55: 468–484. 6187540 10.1016/0013-4694(83)90135-9

[dev70083-bib-0041] Gutierrez‐Galve, L. , A. Stein , L. Hanington , et al. 2019. “Association of Maternal and Paternal Depression in the Postnatal Period With Offspring Depression at Age 18 Years.” JAMA Psychiatry 76: 290–296. 30586134 10.1001/jamapsychiatry.2018.3667 PMC6439819

[dev70083-bib-0042] Gutierrez‐Galve, L. , A. Stein , L. Hanington , J. Heron , and P. Ramchandani . 2015. “Paternal Depression in the Postnatal Period and Child Development: Mediators and Moderators.” Pediatrics 135: e339–e347. 25560437 10.1542/peds.2014-2411

[dev70083-bib-0043] Hajal, N. J. , and S. K. Loo . 2021. “Emerging Biomarkers for Child & Family Intervention Studies: A Review of EEG Studies of Parenting.” Biological Psychology 166: 108200. 34653549 10.1016/j.biopsycho.2021.108200 PMC9256501

[dev70083-bib-0044] Hajcak, G. , J. S. Moser , C. B. Holroyd , and R. F. Simons . 2006. “The Feedback‐Related Negativity Reflects the Binary Evaluation of Good Versus Bad Outcomes.” Biological Psychology 71: 148–154. 16005561 10.1016/j.biopsycho.2005.04.001

[dev70083-bib-0045] Hammen, C. , and P. A. Brennan . 2003. “Severity, Chronicity, and Timing of Maternal Depression and Risk for Adolescent Offspring Diagnoses in a Community Sample.” Archives of General Psychiatry 60: 253–258. 12622658 10.1001/archpsyc.60.3.253

[dev70083-bib-0082] Hart, J. R. , E. E. Coates , and M. A. Smith‐Bynum . 2019. “Parenting Style and Parent‐adolescent Relationship Quality in African American Mother‐adolescent Dyads.” Parenting 19: 318–340.

[dev70083-bib-0087] Hayes, A. F. 2022. Introduction to Mediation, Moderation,and Conditional Process Analysis: a Regression‐based Approach, (3rd ed.). The Guilford Press.

[dev70083-bib-0083] Heaven, P. , and J. Ciarrochi . 2008. “Parental Styles, Gender and the Development of Hope and Self‐esteem.” European Journal of Personality 22: 707–724.

[dev70083-bib-0046] Hill, K. E. , L. Dickey , S. Pegg , A. Dao , K. B. Arfer , and A. Kujawa . 2023. “Associations Between Parental Conflict and Social and Monetary Reward Responsiveness in Adolescents With Clinical Depression.” Research on Child and Adolescent Psychopathology 51: 119–131. 35852700 10.1007/s10802-022-00949-7 PMC9771890

[dev70083-bib-0047] Holroyd, C. B. , G. Hajcak , and J. T. Larsen . 2006. “The Good, the Bad and the Neutral: Electrophysiological Responses to Feedback Stimuli.” Brain Research 1105: 93–101. 16427615 10.1016/j.brainres.2005.12.015

[dev70083-bib-0048] Kendler, K. S. 1996. “Parenting: A Genetic‐Epidemiologic Perspective.” American Journal of Psychiatry 153: 11–20. 10.1176/ajp.153.1.11 8540566

[dev70083-bib-0084] King, K. A. , R. A. Vidourek , and A. L. Merianos . 2016. “Authoritarian Parenting and Youth Depression: Results From a National Study.” Journal of Prevention & Intervention in the Community 44: 130–139. 26939843 10.1080/10852352.2016.1132870

[dev70083-bib-0049] Kujawa, A. , and K. L. Burkhouse . 2017. “Vulnerability to Depression in Youth: Advances From Affective Neuroscience.” Biological Psychiatry: Cognitive Neuroscience and Neuroimaging 2: 28–37. 28497126 10.1016/j.bpsc.2016.09.006 PMC5421558

[dev70083-bib-0050] Kujawa, A. , G. Hajcak , and D. N. Klein . 2019. “Reduced Reward Responsiveness Moderates the Effect of Maternal Depression on Depressive Symptoms in Offspring: Evidence Across Levels of Analysis.” Journal of Child Psychology and Psychiatry 60: 82–90. 29978904 10.1111/jcpp.12944 PMC6296896

[dev70083-bib-0051] Kuzava, S. , G. Nissim , A. Frost , B. Nelson , and K. Bernard . 2019. “Latent Profiles of Maternal Neural Response to Infant Emotional Stimuli: Associations With Maternal Sensitivity.” Biological Psychology 143: 113–120. 30802481 10.1016/j.biopsycho.2019.02.009

[dev70083-bib-0052] Levinson, A. R. , B. C. Speed , B. Nelson , J. N. Bress , and G. Hajcak . 2017. “Authoritarian Parenting Predicts Reduced Electrocortical Response to Observed Adolescent Offspring Rewards.” Social Cognitive and Affective Neuroscience 12: 363–371. 27613780 10.1093/scan/nsw130 PMC5390718

[dev70083-bib-0053] Levinson, A. R. , A. Szenczy , B. D. Nelson , G. Hajcak , and K. Bernard . 2022. “A Biomarker of Maternal Vicarious Reward Processing and Its Association With Parenting Behavior.” Biological Psychology 167: 108240. 34875364 10.1016/j.biopsycho.2021.108240 PMC10575693

[dev70083-bib-0054] Li, Z. , M. L. Sturge‐Apple , and P. T. Davies . 2022. “Family Instability, Parenting, and Child Externalizing Problems: Moderation by Maternal Sympathetic Stress Reactivity.” Development and Psychopathology 35: 1929–1941. 35844100 10.1017/S095457942200058X PMC9845429

[dev70083-bib-0055] Lovejoy, M. C. , P. A. Graczyk , E. O'Hare , and G. Neuman . 2000. “Maternal Depression and Parenting Behavior: A Meta‐Analytic Review.” Clinical Psychology Review 20: 561–592. 10860167 10.1016/s0272-7358(98)00100-7

[dev70083-bib-0056] Luu, P. , D. M. Tucker , D. Derryberry , M. Reed , and C. Poulsen . 2003. “Electrophysiological Responses to Errors and Feedback in the Process of Action Regulation.” Psychological Science 14: 47–53. 12564753 10.1111/1467-9280.01417

[dev70083-bib-0057] Luu, P. , D. M. Tuckera , and S. Makeig . 2004. “Frontal Midline Theta and the Error‐Related Negativity: Neurophysiological Mechanisms of Action Regulation.” Clinical Neurophysiology 115: 1821–1835. 15261861 10.1016/j.clinph.2004.03.031

[dev70083-bib-0058] Merikangas, K. R. , J.‐P. He , D. Brody , P. W. Fisher , K. Bourdon , and D. S. Koretz . 2010. “Prevalence and Treatment of Mental Disorders Among US Children in the 2001–2004 NHANES.” Pediatrics 125: 75–81. 20008426 10.1542/peds.2008-2598 PMC2938794

[dev70083-bib-0059] Mileva‐Seitz, V. , and A. S. Fleming . 2011. “How Mothers Are Born: A Psychobiological Analysis of Mothering.” In Biosocial Foundations of Family Processes, edited by A. Booth , S. M. McHale , and N. S. Landale , 3–34. Springer.

[dev70083-bib-0060] Miltner, W. H. , C. H. Braun , and M. G. Coles . 1997. “Event‐Related Brain Potentials Following Incorrect Feedback in a Time‐Estimation Task: Evidence for a “Generic” Neural System for Error Detection.” Journal of Cognitive Neuroscience 9: 788–798. 23964600 10.1162/jocn.1997.9.6.788

[dev70083-bib-0061] Moses‐Kolko, E. L. , M. S. Horner , M. L. Phillips , A. E. Hipwell , and J. E. Swain . 2014. “In Search of Neural Endophenotypes of Postpartum Psychopathology and Disrupted Maternal Caregiving.” Journal of Neuroendocrinology 26: 665–684. 25059408 10.1111/jne.12183 PMC4353923

[dev70083-bib-0062] O'Doherty, J. , H. Critchley , R. Deichmann , and R. J. Dolan . 2003. “Dissociating Valence of Outcome From Behavioral Control in Human Orbital and Ventral Prefrontal Cortices.” Journal of Neuroscience 23: 7931–7939. 12944524 10.1523/JNEUROSCI.23-21-07931.2003 PMC6740603

[dev70083-bib-0063] Paquette, D. 2004. “Theorizing the Father‐Child Relationship: Mechanisms and Developmental Outcomes.” Human Development 47: 193–219.

[dev70083-bib-0064] Parker, G. , H. Tupling , and L. B. Brown . 1979. “A Parental Bonding Instrument.” British Journal of Medical Psychology 52: 1–10.

[dev70083-bib-0088] Parker, G. 1983. “Development of the Parental Bonding Instrument and the Parent Opinion form.” In Parental Overprotection: a Riskfactor in Psychosocial Development. Grune and Stratton. New York.

[dev70083-bib-0065] Pawluski, J. L. , J. S. Lonstein , and A. S. Fleming . 2017. “The Neurobiology of Postpartum Anxiety and Depression.” Trends in Neurosciences 40: 106–120. 28129895 10.1016/j.tins.2016.11.009

[dev70083-bib-0066] Proudfit, G. H. 2015. “The Reward Positivity: From Basic Research on Reward to a Biomarker for Depression: The Reward Positivity.” Psychophysiology 52: 449–459. 25327938 10.1111/psyp.12370

[dev70083-bib-0067] Reijman, S. , M. J. Bakermans‐Kranenburg , R. Hiraoka , et al. 2016. “Baseline Functioning and Stress Reactivity in Maltreating Parents and at‐Risk Adults: Review and Meta‐Analyses of Autonomic Nervous System Studies.” Child Maltreatment 21: 327–342. 27462035 10.1177/1077559516659937 PMC5058417

[dev70083-bib-0068] Rutherford, H. J. , M. J. Crowley , L. Gao , B. Francis , A. Schultheis , and L. C. Mayes . 2018. “Prenatal Neural Responses to Infant Faces Predict Postpartum Reflective Functioning.” Infant Behavior and Development 53: 43–48. 30314716 10.1016/j.infbeh.2018.09.003

[dev70083-bib-0069] Shafer, K. , B. Fielding , and D. Wendt . 2017. “Similarities and Differences in the Influence of Paternal and Maternal Depression on Adolescent Well‐Being.” Social Work Research 41: 85–96.

[dev70083-bib-0085] Smetana, J. G. 2017. “Current Research on Parenting Styles, Dimensions, and Beliefs.” Current Opinion in Psychology 15: 19–25. 28813261 10.1016/j.copsyc.2017.02.012

[dev70083-bib-0070] Steinberg, L. 2000. “The Family at Adolescence: Transition and Transformation.” Journal of Adolescent Health 27: 170–178. 10.1016/s1054-139x(99)00115-9 10960215

[dev70083-bib-0071] Steinberg, L. , J. S. Silk , and M. H. Bornstein . 2002. “Parenting Adolescents.” In Handbook of Parenting. Routledge.

[dev70083-bib-0072] Sturge‐Apple, M. L. , H. R. Jones , and J. H. Suor . 2017. “When Stress Gets Into Your Head: Socioeconomic Risk, Executive Functions, and Maternal Sensitivity Across Childrearing Contexts.” Journal of Family Psychology 31: 160–169. 27991811 10.1037/fam0000265 PMC6793431

[dev70083-bib-0073] Sturge‐Apple, M. L. , J. H. Suor , and M. A. Skibo . 2014. “Maternal Child‐Centered Attributions and Harsh Discipline: The Moderating Role of Maternal Working Memory Across Socioeconomic Contexts.” Journal of Family Psychology 28: 645–654. 25221969 10.1037/fam0000023 PMC4318501

[dev70083-bib-0074] Trussell, T. M. , W. L. Ward , and N. A. Conners Edge . 2018. “The Impact of Maternal Depression on Children: A Call for Maternal Depression Screening.” Clinical Pediatrics 57: 1137–1147. 29658310 10.1177/0009922818769450

[dev70083-bib-0075] Turney, K. 2011. “Labored Love: Examining the Link Between Maternal Depression and Parenting Behaviors.” Social Science Research 40: 399–415.

[dev70083-bib-0076] Tversky, A. , and D. Kahneman . 1992. “Advances in Prospect Theory: Cumulative Representation of Uncertainty.” Journal of Risk & Uncertainty 5: 297–323.

[dev70083-bib-0077] Volling, B. L. , and N. J. Cabrera . 2019. “Advancing Research and Measurement on Fathering and Child Development: Introducing the Issues and a Conceptual Framework.” Monographs of the Society for Research in Child Development 84: 7–17. 10.1111/mono.12404 31034620

[dev70083-bib-0078] Webb, C. A. , D. G. Dillon , P. Pechtel , et al. 2016. “Neural Correlates of Three Promising Endophenotypes of Depression: Evidence From the EMBARC Study.” Neuropsychopharmacology 41: 454–463. 26068725 10.1038/npp.2015.165 PMC5130121

[dev70083-bib-0079] Weissman, M. M. , O. O. Berry , V. Warner , et al. 2016. “A 30‐Year Study of 3 Generations at High Risk and Low Risk for Depression.” JAMA Psychiatry 73: 970–977. 27532344 10.1001/jamapsychiatry.2016.1586 PMC5512549

[dev70083-bib-0080] Weissman, M. M. , P. Wickramaratne , Y. Nomura , V. Warner , D. Pilowsky , and H. Verdeli . 2006. “Offspring of Depressed Parents: 20 Years Later.” American Journal of Psychiatry 163: 1001–1008. 16741200 10.1176/ajp.2006.163.6.1001

[dev70083-bib-0081] Wrase, J. , T. Kahnt , F. Schlagenhauf , et al. 2007. “Different Neural Systems Adjust Motor Behavior in Response to Reward and Punishment.” Neuroimage 36: 1253–1262. 17521924 10.1016/j.neuroimage.2007.04.001

